# Optimization of drilling processes in panel furniture manufacturing: A case study

**DOI:** 10.1371/journal.pone.0318667

**Published:** 2025-05-12

**Authors:** Guokun Wang, Xiaoli Li, Xianqing Xiong, Siqun Wang

**Affiliations:** 1 College of Furnishings and Industrial Design, Nanjing Forestry University, Nanjing, Jiangsu, China; 2 Center for Renewable Carbon, School of Natural Resources, University of Tennessee, Knoxville, Tennessee, United States of America; 3 Co-Innovation Center of Efficient Processing and Utilization of Forest Resources, Nanjing Forestry University, Nanjing, Jiangsu, China; Manipal Academy of Higher Education, INDIA

## Abstract

The drilling process is a crucial component in the production of panel furniture enterprises; simultaneously, it is also the most complex process. And the furniture enterprise’s transition to intelligent manufacturing lacks effective process optimization. Therefore, this study focuses on optimizing the drilling process in panel furniture. Initially, an analysis of cabinet structures was conducted, followed by data collection on drilling patterns. Based on this data and insights from hole distribution patterns, a novel COING (Coordinate Information Grid) data analysis method was proposed. Subsequently, the application of this method at the Company W, combined with the ARM (Association Rule Mining) method, revealed inconsistencies in drilling process parameters. After proposing and validating solutions in the Company W’s workshop, the findings demonstrated a 14.0% reduction in drilling occurrences and a 3.87% enhancement in drilling efficiency. This study demonstrates the optimization of drilling processes in panel furniture manufacturing.

## Introduction

The rapid development of the customized furniture market has driven a transformation and upgrade of panel furniture enterprises towards intelligent manufacturing [1,2]. These enterprises have introduced a significant amount of high-end equipment and advanced information management systems [3]. This has increased the complexity of production systems, and the introduction of information management systems has also led to challenges such as information silos and redundancy [4,5]. Thus, it presents new demands for production optimization.

Currently, there is considerable literature examining the optimization of furniture issues, with most studies focusing on the positive impacts of optimized furniture layout on daily life [6–9]. However, research on optimizing the furniture production process is relatively limited and fragmented. Ouhimmou et al. optimized the planning of furniture supply chains using a time-decomposition approach [10]. José et al. applied robust optimization methods to production planning within furniture environments [11]. Oliveira et al. optimized the furniture cutting process from the perspective of information systems [12]. Nyemba utilized machine distance matrices to map and optimize the process flow of a furniture manufacturing company in Zimbabwe [13] and Nouri optimized mixed assembly lines for wooden furniture through computer simulation [14].

The production of panel furniture primarily involves four processes: cutting, edge banding, drilling, and packaging. Due to its high degree of customization and complex cabinet structures, panel furniture exhibits high degrees of freedom, massive quantities, and varied types of hole positions. The drilling process, critical in determining both production line efficiency and product appearance and stability, is thus the most complex procedure in panel furniture production.

However, current research on the drilling process of panel furniture remains limited, primarily focusing on cabinet connector components. For instance, Krzyżaniak analyzed the strength and stiffness of a concealed detachable joint connector, while Kuşkun conducted experimental and numerical analyses on the installation force of expanding dowels used in furniture connections [15,16]. Kasal examined the connection of L-shaped corner joints in panel furniture with auxiliary dowels [17]. Only Wang et al. briefly mentioned the optimization of the drilling process within scheduling algorithms [18].

The above literature review indicates a lack of systematic optimization methods for panel furniture and a limited focus on the drilling process. Therefore, this paper aims to optimize the drilling process in panel furniture enterprises. After analyzing cabinet structures and collecting data on drilling patterns, a novel data analysis method known as the Coordinate Information Grid (COING) has been developed. This method was applied at the Company W, a prominent panel furniture manufacturer in China, in conjunction with the Association Rule Mining (ARM) technique, effectively realizing the optimization of the drilling process.

## Methods and experimental

### Basic structure of panel furniture

**Fig 1:**
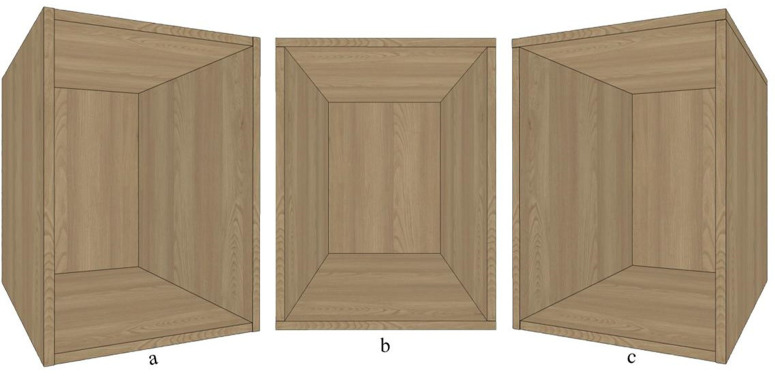
Three types of typical connection structures: (a) side panels covering top and bottom panels, (b) top and bottom panels covering side panels, and (c) top panel covering side panels and side panels covering bottom panel.

The fundamental structure of panel furniture consists primarily of a frame structure composed of a top panel, bottom panel, left side panel, right side panel, and back panel. There are typically three types of connection structures: side panels covering top and bottom panels, or top and bottom panels covering side panels, or top panel covering side panels and side panels covering bottom panel (Fig 1). The connection between the top panel, bottom panel, and/or side panels is usually achieved through eccentric connecting components [19]. The connection method between the back panel and the top, bottom, and side panels depends on the thickness of the back panel. Thin back panels with thicknesses of 3 mm, 5 mm, 9 mm, or 12 mm are typically connected through slotting and embedding, while thick back panels with thicknesses of 16 mm or 18 mm use the same eccentric connecting components.

**Fig 2:**
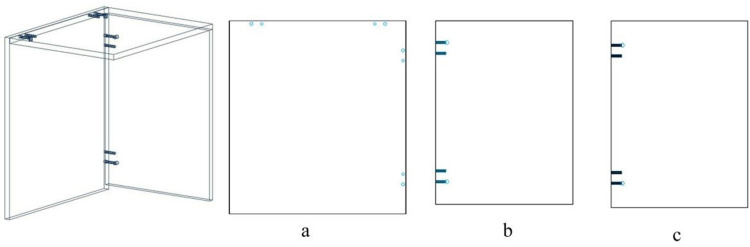
Side panel covering top panel with a thick back panel: (a) side panels, (b) top panel, and (c) thick back panel.

**Fig 3:**
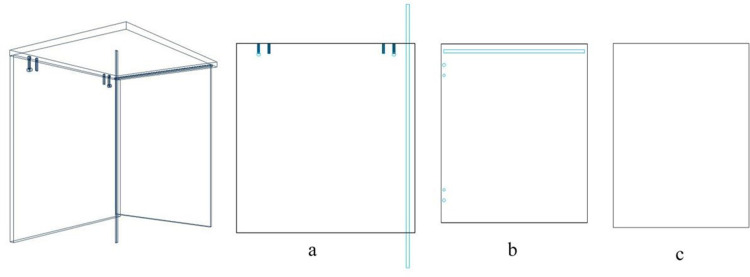
Top panel covering side panel with a thin back panel: (a) side panels, (b) top panel, and (c) thin back panel.

Taking two scenarios as examples, namely side panel covering top panel with a thick back panel (Fig 2) and top panel covering side panel with a thin back panel (Fig 3), the cabinet structure and hole processing of the basic panel components are illustrated.

### Collection of drilling data

Panel furniture enterprises aiming to develop towards intelligent manufacturing require detailed information about panels and drilling drawings for the drilling process. The basic information includes N orders {O1, O2, O3, ..., On}, where each order consists of M panels {P1, P2, P3,..., Pm} and X drawings {D1, D2, D3, ..., Dx}. Additionally, each panel in an order is characterized by its dimensions, hole information, and groove information (as shown in Table 1).

**Table 1:** Panel information.

Dimensions	Abbreviation	Hole information (HI)	Abbreviation	Slot information (SI)	Abbreviation
Length	LE	Front Hole	FH	Front Slot	FS
Width	WI	Back Hole	FH	Back Slot	BS
Thickness	TH	Double Hole	DH	Double Slot	DS
		Horizontal Hole	HH	Horizontal Slot	HS

Using NA (Name) denotes the name of the panel. Thus, the basic information P (Panel) for each panel can be described as a set consisting of the following eight fundamental parameters:


P = {NA, LE, WI, TH, HI, SI, Dx, D2x};

Since panels are often processed on both sides, there may be two drawings corresponding to a single panel. The reverse drawing information D2x corresponds to the panel information P.


$$Dix=\left\{ \begin{array}{c} D2x\;Have\;a\;reverse\;side\;drawing\;,\;it\;will\;be\;indicated\;as\;"D2x"\\ Null\;No\;reverse\;side\;drawing,\;it\;will\;be\;left\;blank\; \end{array}\right.\;$$

Therefore, the drilling information for an order on can be expressed as an M *  8 matrix Z.


$$Z=\left(\begin{array}{ccc} NA1 & 1500 & 550\;\\ NA2 & 564 & 549\\ \begin{array}{c} \ldots \ldots \\ \ldots \ldots \\ NAm \end{array} & \begin{array}{c} \ldots \ldots \\ \ldots \ldots \\ 664 \end{array} & \begin{array}{c} \ldots \ldots \\ \ldots \ldots \\ 519 \end{array} \end{array}\begin{array}{ccc} 18 & FH & FS\;D1\;Null\\ 18 & FH & BS\;D2\;D3\\ \begin{array}{c} \ldots \ldots \\ \ldots \ldots \\ 25 \end{array} & \begin{array}{c} \ldots \ldots \\ \ldots \ldots \\ BH \end{array} & \begin{array}{c} \ldots \ldots \\ \ldots \ldots \\ \;\backslash \;Dx\;D2x \end{array} \end{array}\right)$$

The processing methods for panels primarily include vertical drilling (VD), horizontal drilling (HD), grooving (GR), and milling (MI), determined by the assembly method of panel furniture.

The drawing information for panels includes drawing numbers, panel dimensions, and processing details. Since the drawing number and dimension details are already captured in set Z, the drawing information can be represented as collection G, which includes Dx, Dix, and processing details.


G=(D1,VD1,X−axis coordinate,Y−axis coordinate,Diameter,Depth;D1,VD2,X−axis coordinate,Y−axis coordinate,Diameter,Depth;D1,HD1,X−axis coordinate,Y−axis coordinate,Z−axis coordinate,Diameter,DepthD1,HD2,X−axis coordinate,Y−axis coordinate,Z−axis coordinate,Diameter,DepthD1,Groove,Starting X−axis coordinate,Starting Y−axis coordinate,Ending X−axis coordinate,Ending Y−axis coordinate,Depth,Width;……………………………………………………………………; D2,VD1,X−axis coordinate,Y−axis coordinate,Diameter,Depth;D2,VD2,X−axis coordinate,Y−axis coordinate,Diameter,Depth;D2,Milling,Cut X−axis midpoint,Ending X−axis coordinate,Milling cut length,Milling cut width,Radial radius,Depth;……………………………………………………………………; Dix,……………………………………………………………………;)

By transforming panel and drawing information into matrix Z and set G, it becomes possible to discern all panel details and corresponding drawing information within order O, thereby facilitating the collection and representation of drilling data.

### Coordinate information grid method

After completing data collection, it is necessary to conduct mining and analysis. Methods for data mining analysis include classification, clustering, association rule mining, and prediction [20].

Classification aims to extract models from datasets that describe fundamental characteristics of data classes, enabling the classification of each object into a known data class [21]. Clustering involves dividing a dataset into different classes or clusters according to specific criteria, maximizing the similarity of data objects within the same cluster and maximizing the dissimilarity between objects in different clusters [22]. Essentially, clustering seeks to group similar data together while keeping different data apart. Association rule mining (ARM) analysis is a descriptive method for analyzing data that discovers interesting associations or relationships within a dataset [23]. Prediction analysis entails using information obtained from large datasets to forecast and estimate future outcomes.

In order to ensure the structural stability of panel furniture, panels are typically oriented horizontally or vertically. Therefore, a group of holes to be processed must consist of at least two holes, and holes within the same group generally lie along the same horizontal or vertical line on the drawing, sharing identical X or Y coordinates. Thus, the information regarding hole positions on panels is not entirely discrete but follows a certain regularity. Using a clustering approach for analyzing hole positions is quite appropriate.

**Fig 4:**
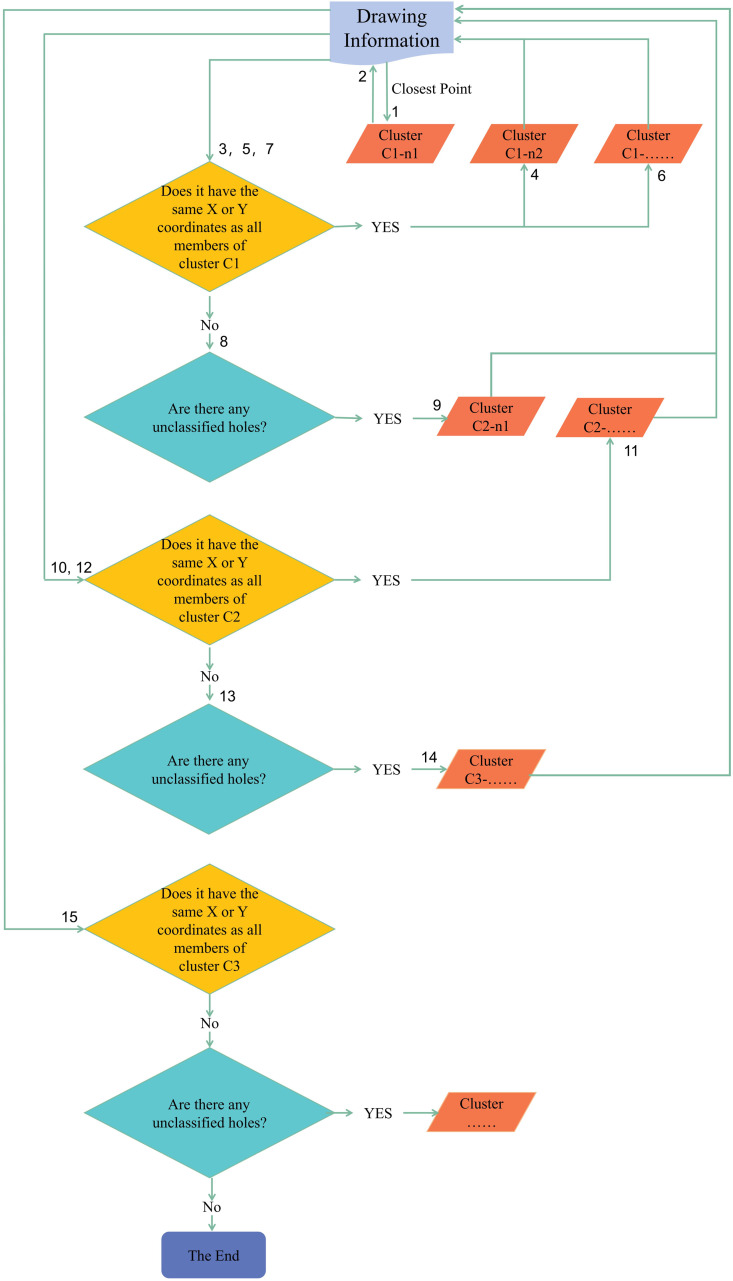
The coordinate grid method.

Common clustering methods include grid-based algorithms, such as the STING (Statistical Information Grid) method, which divides spatial regions into rectangular units, and algorithms like k-means, k-medoids, BIRCH, CURE, and CHAMELEON, which partition space using hierarchical and recursive methods. However, since the hole positions in panel furniture manufacturing follow specific patterns due to design and functional requirements, this paper proposes a novel method called the Coordinate Information Grid (COING) for analyzing hole positions on panels based on the clustering analysis [24,25]. The specific steps are as follows, and the number next to the arrow indicates the specific steps (Fig 4):

a. Select the panel P in order O as the object for analysis and read the corresponding drawing information Dix based on the panel drawing information;
b. Use set G to classify the drawing information according to different processing rules;
c. Select the point closest to the origin as the first member n1 of cluster C1;
d. Search for points n2 with the same X or Y-axis coordinate parameters as the members of cluster C1, and update the coordinates of n2 to cluster C1;
e. Search for points n3 with the same X or Y-axis coordinate parameters as the updated members of cluster C1, and update the new coordinates to cluster C1;
f. Repeat step (5) until no points that meet the criteria can be found in the drawing.
g. Select the point closest to the origin outside of cluster C1 as the first member n1 of the second cluster C2;
h. Repeat step (5) until no points that meet the criteria can be found in the drawing.
i. Repeat steps (7) and (8) until all points are included in the clusters;
j. Handle abnormal data, such as marking the panel if there is only one point in the cluster, and conducting targeted analysis;
k. Analyze the number, distance, processing parameters, distribution type, etc. of the hole positions in the same cluster.

The COING method aggregates holes on panels that share common X or Y axis coordinates. This approach enables a clear observation of spatial relationships between hole positions, facilitates the discovery of hidden correlations among hole placements, and identifies potential issues within the drilling process.

### Experimental study at Company W

The Company W is a prominent publicly listed company in Nanjing, China, widely recognized as a leading representative of the panel furniture manufacturing industry. The company has introduced advanced drilling equipment from Germany’s HOMAG company and implemented a new Manufacturing Execution System (MES) in its workshops. However, the expected efficiency improvements in the drilling process have not been realized. Therefore, this research attempts to collect panel and drawing information, and employs the COING method to analyze drilling process data, aiming to achieve optimization for the company.

### Panel and drawing data

A total of 41,689 panels produced by Company W in one day were randomly selected from the company’s ERP system. The panel information was summarized using matrix Z. To facilitate storage and access, the information exported from the MES system for that day was stored in an EXCEL spreadsheet, and a portion of the data is shown in Table 2.

**Table 2:** Partial panel information of Company W.

NA	LE	WI	TH	HI	SI	Dx	Dix
Right side panel - with light groove	2276	400	18	FH	FS	SO03063233109181	NULL
Left side panel - with light groove	2276	400	18	FH	FS	SO03063233101904	NULL
Bottom panel	782	379	18	BH	FS	SO03063233102281	SO03063233202284
Top panel	984	459	18	FH	BS	SO03062730100221	SO03062730200224
Bottom panel	984	459	18	DH	FS	SO03062730100301	SO03062730200304
Right side panel	2228	525	18	DH	DS	SO03062733107001	SO03062733207004
Left side panel	2304	571	18	FH	FS	SO03062740100264	NULL
Right side panel	2254	550	25	FH	FS	SO03062740100341	NULL
Kickboard	875	80	18	HH	\	SO03062733107241	NULL
Back pull strip	875	80	18	FH	\	SO03062733107481	NULL
Thin back panel	974	574	9	FH	\	SO03062733107491	NULL

Company W’s standard drawing file format for drilling processes is MPR. Through analysis of this drawing format, the required information is extracted according to set G. Due to the large amount of data, a Python code was written to extract and store the data in an SQL Server database, and a portion of the data is shown in Table 3.

**Table 3:** Partial drawing data of Company W.

Panel Code	Processing Type	Parameter 1	Parameter 2	Parameter 3	Parameter 4	Parameter 5	Parameter 6
SO03063233109181	VD	34	68	15	14	NULL	NULL
SO03063233109181	VD	34	484	15	14	NULL	NULL
SO03063233109181	VD	747	68	15	14	NULL	NULL
SO03063233109181	VD	747	484	15	14	NULL	NULL
SO03063233109181	HD	0	68	9	8	32	NULL
SO03063233109181	HD	0	484	9	8	32	NULL
SO03063233109181	HD	0	100	9	8	22	NULL
SO03063233109181	HD	0	452	9	8	22	NULL
SO03063233109181	HD	781	68	9	8	32	NULL
SO03063233109181	HD	781	484	9	8	32	NULL
SO03063233109181	HD	781	100	9	8	22	NULL
SO03063233109181	HD	781	452	9	8	22	NULL
SO03063233109181	GR	-120	901	526	526	10	5.5
SO03063233109181	MI	390	450	680	6	3	5
……	……	……	……	……	……	……	……

### COING clustering analysis

**Fig 5:**
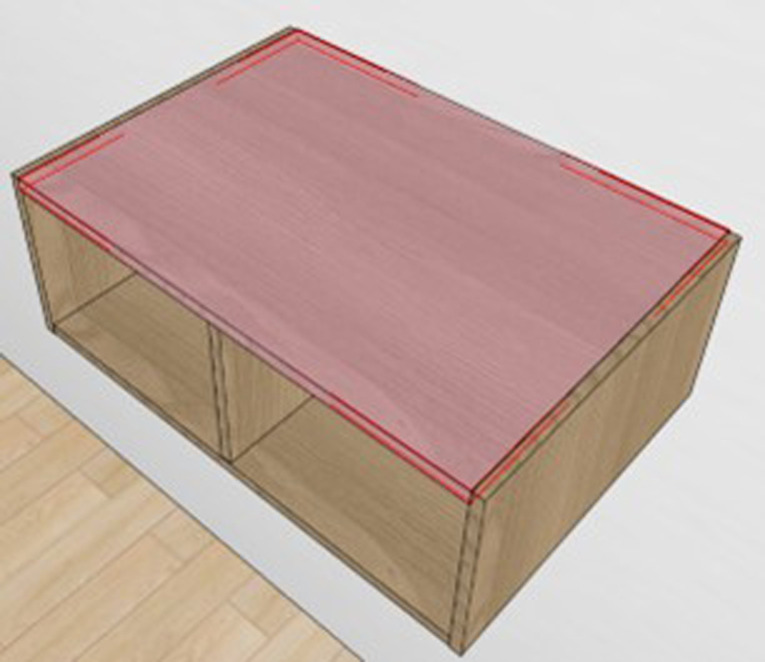
The top panel of the cabinet.

Taking a certain top panel as an example (Fig 5).

The information of the top plate can be represented as:

P =  {Top panel, 764, 550, 18, FH, \, SO2024082610006, NULL};

The drawing information of the top plate can be represented as:


$$G=\left(\begin{array}{c} \begin{array}{c} \begin{array}{c} SO2024082610006,VD1,34,68,15,14;\\ SO2024082610006,VD2,34,484,15,14; \end{array}\\ SO2024082610006,VD3,730,68,15,14; \end{array}\\ SO2024082610006,VD4,730,484,15,14;\\ SO2024082610006,VD5,382,68,10,10;\\ SO2024082610006,VD6,382,100,8,10;\\ SO2024082610006,VD7,382,452,8,10;\\ SO2024082610006,VD8,382,484,10,10;\\ SO2024082610006,HD1,0,68,9,8,32;\\ SO2024082610006,HD2,0,100,9,8,22;\\ SO2024082610006,HD3,0,452,9,8,22;\\ SO2024082610006,HD4,0,484,9,8,32;\\ SO2024082610006,HD5,764,68,9,8,32;\\ SO2024082610006,HD6,764,100,9,8,22;\\ SO2024082610006,HD7,764,452,9,8,22;\\ SO2024082610006,HD8,764,484,9,8,32; \end{array}\right)$$

The specific COING analysis of the top panel is as follows:

a. Find the point closest to the origin as a horizontal hole, which can be represented as:

GC1n1 = {horizontal hole n1, 0, 68, 9, 8, 32}

Include it as a member n1 in the first cluster C1;

b. Based on the X coordinate 0 of GC1n1, search for points with an X coordinate of 0:

GC1n2 = {horizontal hole n2, 0, 100, 9, 8, 22};

GC1n3 = {horizontal hole n3, 0, 452, 9, 8, 22};

GC1n4 = {horizontal hole n4, 0, 484, 9, 8, 32};

Include them in cluster C1;

c. Based on the updated C1, search for four points with the same Y coordinate:

GC1n5 = {horizontal hole n2, 764, 68, 9, 8, 32};

GC1n6 = {horizontal hole n2, 764, 100, 9, 8, 22};

GC1n7 = {horizontal hole n2, 764, 452, 9, 8, 22};

GC1n8 = {horizontal hole n2, 764, 484, 9, 8, 32};

Include them in cluster C1;

d. When no points that meet C1 are found, find the point closest to the origin outside cluster C1, which is a vertical hole and can be represented as:

GC2n1 = {vertical hole n1, 34, 68, 15, 14};

Include it as a member n1 in the second cluster C2;

e. Based on the X coordinate 34 of GC2n1, search for points with an X coordinate of 34:

GC2n2 = {vertical hole n2, 34, 484, 15, 14};

Include it in cluster C2;

f. Based on the updated C2, search for two points with the same Y coordinate:

GC2n3 = {vertical hole n3, 730, 68, 15, 14};

GC2n4 = {vertical hole n4, 730, 484, 15, 14};

Include them in cluster C2;

g. When no points that meet C2 are found, find the point closest to the origin outside clusters C1 and C2, which is a vertical hole and can be represented as:

GC3n1 = {vertical hole n1, 382, 68, 10, 10};

Include it as a member n1 in the third cluster C3;

h. Based on the updated C3, search for three points with the same X coordinate:

GC3n2 = {vertical hole n2, 382, 100, 8, 10};

GC3n3 = {vertical hole n3, 382, 452, 8, 10};

GC3n4 = {vertical hole n4, 382, 484, 10, 10};

Include them in cluster C3;

i. All hole positions of the panel are included in the corresponding clusters, and no abnormal points are found.
j. The results indicate that cluster C1 comprises 8 horizontal holes, C2 contains 4 vertical holes, and C3 also contains 4 vertical holes. The top panel categorized into clusters C1, C2, and C3 can be hierarchically displayed, as shown in Fig 6.

**Fig 6:**
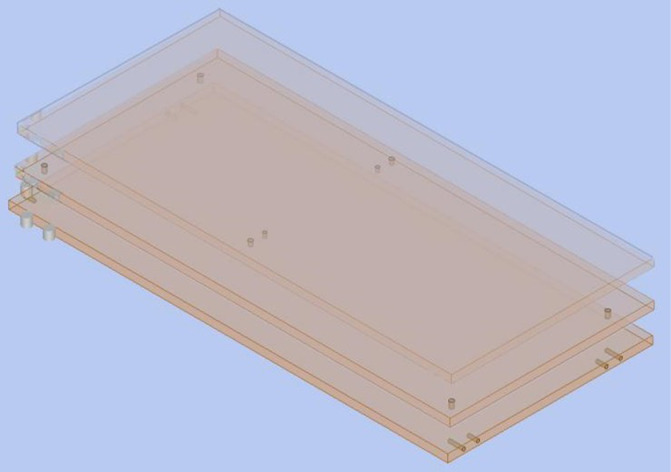
Results of COING clustering analysis.

The processing parameters for vertical and horizontal holes can be represented as diameter *  depth (φ *  D).

Based on the COING method, a data analysis was conducted for each panel, and each grid was divided into different levels. Through analysis of 41,689 panels with Python code, it was observed that horizontal holes of sizes 8 * 32 and 8 * 22 frequently appear within the same cluster. Vertical holes of sizes 5 * 9, 8 * 10, 10 * 10, 15 * 14, 18 * 13, and 35 * 14 appear in the same cluster. These frequently occurring hole dimensions are closely related to the connectors used, which are determined by the assembly process and manufacturing techniques.

### Association rule mining analysis

Although Company W employed the COING method to classify hole position data, it appears that the resulting dataset lacked granularity. To further investigate the relationships between hole positions, Company W employed ARM methods for further analysis. The ARM method can discover interesting associations or relationships within the drilling data.

Association rules are typically represented as implications X → Y, where X ⊂ I denotes the antecedent and Y ⊂ I denotes the consequent, with X ∩ Y=∅. Both X and Y are itemset in the dataset (D), and I represents the set of all item sets. The rule implies that if itemset X appears in D, it leads to the occurrence of Y with a certain probability, and X and Y have no intersection [26,27]. This probability of occurrence is known as the confidence, which reflects the strength of the relationship between itemsets X and Y.

Company W employs the ARM method to analyze the data following these steps:

a. Based on the processing parameters of different panels, generate the set of all itemsets. In this context, the itemsets comprise high-frequency hole configurations identified through COING analysis, which include:

IVD = {5*9,8*10,10*10,15*14,18*13,35*14};

IHD = {8*22,8*32}.

b. Calculate the support of itemsets. The support of an itemset defined as the probability of itemset X occurring in D, where X represents a drilling parameter within IVD and IHD, can be expressed as:

Support(X)=count(X)/|D|;

From the SQL Server database, the counts of panels containing IVD and IHD respectively are obtained. By computing the support of each itemset within IVD and IHD, it can derive: IVDSupport(5 * 9) = 2349/41689 = 5.635%;

IVDSupport(8*10)=14818/41689=35.544%;

IVDSupport(10*10)=12423/41689=29.799%;

IVDSupport(15*14)=12423/41689=29.799%;

IVDSupport(18*13)=4518/41689=10.837%;

IVDSupport(35*14)=89/41689=0.213%;

IHDSupport(8*22)=14818/41689=35.544%;

IHDSupport(8*32)=12423/41689=29.799%.

c. Define frequent itemsets. By defining a minimum support threshold minsup, itemsets T satisfying Support (X)≥minsup can be filtered and designated as frequent itemsets. Setting the minimum support minsup=20%, the resulting frequent itemsets IVDT1and IHDT1 for IVD and IHD respectively are:

IVDT1 = {8*10,10*10,15*14};

IHDT1 = {8*22,8*32}.

d. Generate candidate itemsets. For any frequent itemset T in D, generate all its non-empty subsets t⊂T; From the frequent itemset IVDT1, the set of candidate itemsets IVDt1 is produced as follows:

IVDt1={{8*10,10*10}, {8*10,15*14}, {10*10,15*14}}.

IHDT1 has no candidate itemsets to generate.

e. Calculate the support for each candidate item set in IVDt1, which represents the probability of a panel containing two specific drilling parameters simultaneously:

IVDt1Support(8*10,10*10)=11719/41689=28.111%;

IVDt1Support(8*10,15*14)=3612/41689=8.664%;

IVDt1Support(10*10,15*14)=3612/41689=8.664%.

Since IHDT1 has only two hole position parameters for frequent item sets, therefore, support analysis can proceed directly:

IHDt1Support(8*22,8*32)=11719/41689=28.111%.

f. Generate candidate itemsets again. Due to the minimum support threshold minsup=20%, the generated frequent itemsets IVDT2 and IHDT2 are as follows:

IVDT2={8*10,10*10};

IHDT2={8*22,8*32}.

g. Determine strong association rules by analyzing the confidence of the association rules. Confidence is the probability of the consequent Y occurring given that the antecedent X has occurred. It can be calculated as:

Confidence(X→Y)=Support(X→Y)/Support(X);

h. Therefore, the confidence of IVDT2 and IHDT2 is:

Confidence(8*10→10*10)==Support(8*10→10*10)/Support(8*10)=11719/14818=79.086%;

Confidence(8*22→8*32)==Support(8*22→8*32)/Support(8*22)=11719/14818=79.086%.

Set mincon = 50%. The results of confidence are both greater than mincon, so IVDT2 and IHDT2 are the frequent item sets sought, and the strong association rules are:

8 * 10 → 10 * 10 [Support = 28.111%, Confidence = 79.086%];

8*22→8*32 [Support=28.111%, Confidence=79.086%].

## Results and discussion

### Result analysis

Based on the analysis results obtained from COING and ARM methods, there exists a strong association between the vertical holes with parameters 8 * 10 and 10 * 10, as well as the horizontal holes with parameters 8 * 22 and 8 * 32.

Furthermore, the confidence values for these associations are also identical. Based on the results of ARM, particular attention was given to the observation of the actual panel processing procedure, revealing that the drilling parameters for 10 * 10 vertical holes and 8 * 32 horizontal holes are primarily required for the eccentric connectors used in cabinet assembly. Additionally, the drilling parameters for 8 * 10 vertical holes and 8 * 22 horizontal holes are required for the wooden tenons used in conjunction with the connectors.

By examining the multi-layered drawings generated through COING methods, it is evident that the spacing between holes 8 * 22 and 8 * 32, as well as between holes 8 * 10 and 10 * 10, is consistently 32 mm. This is because the Company W follows the 32 mm principle when setting the linear proportion for drilling. The spacing between vertical and horizontal holes, which is used in conjunction with the dowels, adheres to the 32 mm system. The proper application of the 32 mm system enables single-axis movement and facilitates the efficient production of multi-hole panels.

However, achieving the desired outcome not only requires a hole spacing of 32 mm but also consistent processing parameters for the hole positions. Although the Company W has designed a drilling scheme based on the 32 mm system, the inconsistency in processing parameters has resulted in inefficiencies in the drilling process.

### Optimization solution

**Fig 7:**
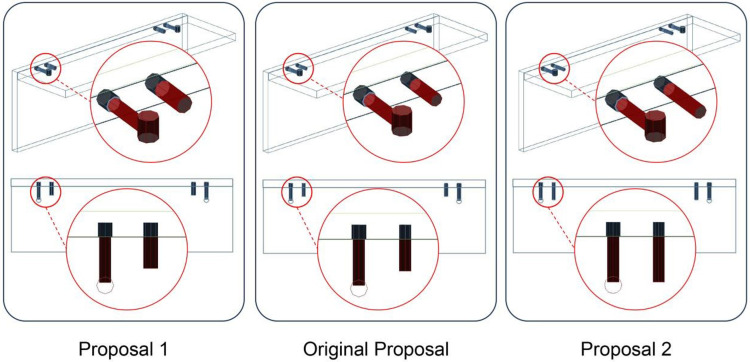
Comparison of two proposals with the original proposal.

Two optimization solutions are proposed for the issues encountered by the Company W: Firstly, changing the diameter of the wooden tenon to 10 mm would achieve consistency in the parameters of the vertical holes in the side panel. Secondly, altering the length of the tenon from 30 mm to 40 mm would ensure consistency in the parameters of the horizontal holes in the top panel (Fig 7).

The Company W employs drilling equipment from German company HOMAG, including the ABL220 and PTP160 devices. The main spindle drill boxes can accommodate multiple vertical and horizontal drill bits, with a spacing of 32 mm between each drill box. This satisfies the requirements for using the 32 mm system.

Therefore, after evaluating both solutions and considering the increased cost of larger tenon sizes and tool wear, the Company W has decided to implement the second solution: changing the length of the tenon from 30 mm to 40 mm. This adjustment aligns the parameters of the tenon’s horizontal holes with those of the connecting hardware’s horizontal holes. By fully utilizing the advantages of the equipment and the 32 mm system, the drilling equipment can achieve one-time movement of the main spindle, resulting in both horizontal holes being made simultaneously.

Based on randomly selected production data from the Company W’s ERP system over a period of 26 days, it was found that the company produces an average of 33,609 panels per day, with an average of 246,718 boreholes processed daily. The number of tenons required to meet the 32 mm system’s spacing in conjunction with the connecting hardware is 34,785. By upgrading the tenon to 8 * 40 mm, the daily number of boreholes can be reduced by 34,785, theoretically resulting in an overall decrease of 14.0% in borehole quantity.

### Effect validation

**Fig 8:**
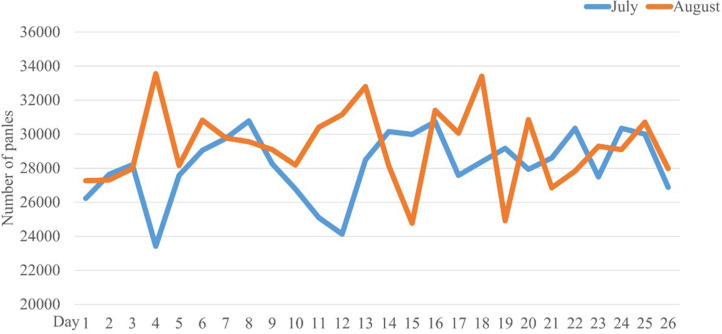
Comparison of drilling process panel data for July and August.

The Company W optimized the process using the second solution on August 1, 2023. Under consistent conditions for equipment utilization rate, personnel count, equipment quantity, and production time, production data from the MES system for the production lines utilizing the optimized scheme were retrieved for comparative analysis (Fig 8).

The Fig 8 illustrates the drilling data for the Company W in July and August 2023. Both months had 26 production days. In July, the drilling process processed a total of 732,953 panels, whereas in August, 761,315 panels were processed. Post-optimization, the overall efficiency of the drilling process improved by 3.87%. Empirical validation confirmed that the optimization effectively enhanced the drilling process by reducing the number of drilling operations.

## Conclusions

This study addresses the lack of systematic optimization methods for the drilling process in panel furniture enterprises. By developing the Coordinate Information Grid (COING) and implementing it at the Company W alongside the Association Rule Mining (ARM) technique, we identified inconsistencies in drilling process parameters. The optimized results demonstrated a significant 14.01% reduction in drilling occurrences and a 3.87% increase in drilling efficiency. The implications of this study suggest that similar methodologies could be applied to other manufacturing processes to identify inefficiencies and drive improvements.

This research has several limitations. It does not account for factors such as equipment configuration, workshop layout, and production scheduling. Furthermore, the focus on a single company and equipment restricts the generalizability of the findings. Future research should investigate these aspects and validate the results in different contexts. Additionally, exploring further optimization techniques such as the use of AI, machine learning, and digital twin technologies, along with their long-term impacts on panel furniture manufacturing, could yield greater efficiencies and potentially promote the development of panel furniture companies towards intelligent manufacturing.

## Supporting information

S1 File XXX. (ZIP)
